# Postoperative rendezvous endoscopic retrograde cholangiopancreaticography as an option in the management of choledocholithiasis

**DOI:** 10.1007/s00464-019-07272-1

**Published:** 2019-11-25

**Authors:** Eva-Lena Syrén, Gabriel Sandblom, Staffan Eriksson, Arne Eklund, Bengt Isaksson, Lars Enochsson

**Affiliations:** 1 Department of Surgical Sciences, Uppsala University, 751 35 Uppsala, Sweden; 2 Centre of Clinical Research, Västmanland Hospital, Västerås, Sweden; 3 Department of Clinical Science and Education Södersjukhuset, Karolinska Institute, Stockholm, Sweden; 4 Department of Surgery, Södersjukhuset, Stockholm, Sweden; 5 Sunderby Research Unit, Department of Surgical and Perioperative Sciences, Surgery, Umeå University, Umeå, Sweden

**Keywords:** Rendezvous ERCP, Choledocholithiasis, Complications

## Abstract

**Background:**

Rendezvous endoscopic retrograde cholangiopancreaticography (ERCP) is a well-established method for treatment of choledocholithiasis. The primary aim of this study was to determine how different techniques for management of common bile duct stone (CBDS) clearance in patients undergoing cholecystectomy have changed over time at tertiary referral hospitals (TRH) and county/community hospitals (CH). The secondary aim was to see if postoperative rendezvous ERCP is a safe, effective and feasible alternative to intraoperative rendezvous ERCP in the management of CBDS.

**Methods:**

Data were retrieved from the Swedish registry for cholecystectomy and ERCP (GallRiks) 2006–2016. All cholecystectomies, where CBDS were found at intraoperative cholangiography, and with complete 30-day follow-up (n = 10,386) were identified. Data concerning intraoperative and postoperative complications, readmission and reoperation within 30 days were retrieved for patients where intraoperative ERCP (n = 2290) and preparation for postoperative ERCP were performed (n = 2283).

**Results:**

Intraoperative ERCP increased (7.5% 2006; 43.1% 2016) whereas preparation for postoperative ERCP decreased (21.2% 2006; 17.2% 2016) during 2006–2016. CBDS management differed between TRHs and CHs. Complications were higher in the postoperative rendezvous ERCP group: Odds Ratio [OR] 1.69 (95% confidence interval [CI] 1.16–2.45) for intraoperative complications and OR 1.50 (CI 1.29–1.75) for postoperative complications. Intraoperative bleeding OR 2.46 (CI 1.17–5.16), postoperative bile leakage OR 1.89 (CI 1.23–2.90) and postoperative infection with abscess OR 1.55 (CI 1.05–2.29) were higher in the postoperative group. Neither post-ERCP pancreatitis, postoperative bleeding, cholangitis, percutaneous drainage, antibiotic treatment, ICU stay, readmission/reoperation within 30 days nor 30-day mortality differed between groups.

**Conclusions:**

Techniques for management of CBDS found at cholecystectomy have changed over time and differ between TRH and CH. Rendezvous ERCP is a safe and effective method. Even though intraoperative rendezvous ERCP is the preferred method, postoperative rendezvous ERCP constitutes an acceptable alternative where ERCP resources are lacking or limited.

Laparoscopic cholecystectomy (LC) has become the gold standard worldwide for treatment of gallstone disease. In Sweden about 13.000 cholecystectomies are performed each year and the vast majority of these with minimally invasive surgery [1]. Intraoperative cholangiography (IOC), is routinely performed in Sweden in order to clarify the anatomy of the biliary tree and has also proved to be an effective method to detect common bile duct stones (CBDS) which are found in 10–15% of cases [1–5].

In recent years intraoperative rendezvous endoscopic retrograde cholangiopancreaticography (ERCP) has been established as an alternative method to treat common bile duct stones discovered during laparoscopic cholecystectomy. This laparo-endoscopic rendezvous (LERV) technique was first described in 1993 by Deslandres et al. [6] and has been shown to have a high rate of CBD stone clearance and a lower complication rate, particularly post-ERCP pancreatitis, compared to conventional ERCP [7–15]. This may be due to the facilitated access to the common bile duct with less manipulation and trauma to the papilla Vateri.

An alternative to the single-session intraoperative ERCP procedure is the postoperative rendezvous ERCP technique, in which the antegrade transcystic guidewire is passed into the duodenum and anchored to the cystic duct with laparoscopic clips. The opposite end of the guidewire is then deviated through the abdominal wall and attached to the skin, leaving the guidewire in situ. The cholecystectomy procedure is completed and the rendezvous ERCP conducted within a few days afterwards using the guidewire to help cannulate the bile duct.

Intraoperative rendezvous ERCP has been recommended as the method of choice rather than postoperative rendezvous ERCP due to lower morbidity, lower costs and shorter hospital stay [16–19]. Nevertheless, the extended operation time and limited access of endoscopic expertise is associated with organizational and logistic challenges with this technique [8, 9, 14]. There are several units in Sweden where cholecystectomies are performed without ERCP resources available. Furthermore, in most of the units where ERCP is an established method for management of common bile duct stones during cholecystectomy, there is no endoscopic expertise available during evenings, week-ends and sometimes not even on a regular basis during weekdays [1].

The primary aim of this nation-wide population-based study was to assess how different techniques for the management of CBDS clearance have changed over time at TRHs and CHs. The secondary aim was to see if postoperative rendezvous ERCP is a safe, effective and feasible alternative to intraoperative rendezvous ERCP in the management of CBDS clearance and complications.

## Materials and methods

The study was based on a cohort of prospectively registered data from GallRiks (The Swedish National Quality Registry for Gallstone Surgery and ERCP) 2006–2016.

GallRiks started 1st of May 2005 and covers about 90% of all cholecystectomies and ERCPs in Sweden. All ERCPs are registered-, together with patient- and procedure-related data. All intra- and postoperative complications are registered, and the completeness of 30-day follow-up of postoperative complications, including post-ERCP pancreatitis (PEP), is approximately 95%. PEP is defined according to the Cotton criteria [20]. GallRiks is regularly externally validated [21, 22].

In the case of choledocholithiasis found at cholecystectomy, data were registered in GallRiks as one of the following treatment alternatives: “open choledochotomy”; “transcystic stone extraction”; “flushed or manipulated stones”; “laparoscopic choledochotomy”; “intraoperative ERCP/rendezvous ERCP”; “preparation for postoperative ERCP/rendezvous ERCP”; or “no further procedure”.

**Fig. 1:**
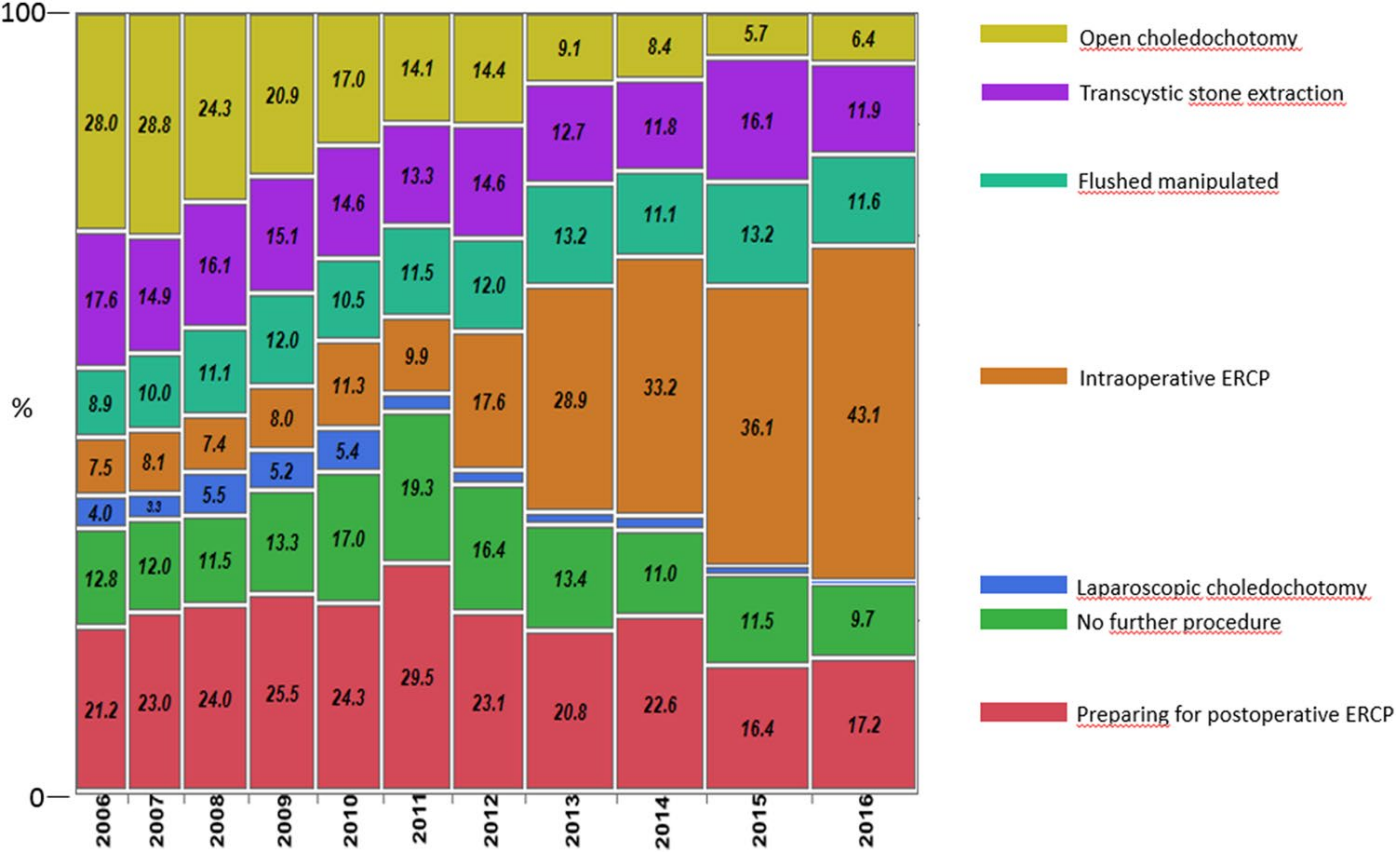
Alternative techniques for management of CBDS found at cholecystectomy in Sweden 2006–2016

**Fig. 2:**
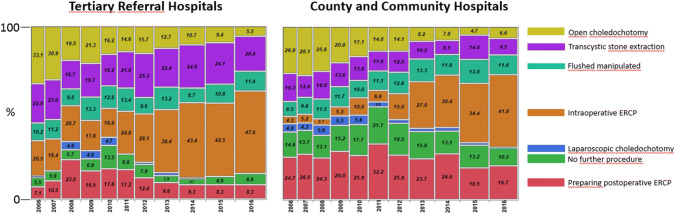
Frequency of intraoperative ERCP and preparing postoperative ERCP as alternatives to treat CBDS discovered during cholecystectomy at Tertiary Referral Hospitals compared with County and Community Hospitals in Sweden 2006–2016

Data on methods used to treat CBDS during scheduled and acute cholecystectomies at tertiary referral hospitals and county and community hospitals were collected (Figs. 1, 2). In Sweden there are seven university hospitals/tertiary referral hospitals and 65 county and community hospitals.

The primary outcome of this study was changes in techniques used for management of common bile duct stone (CBDS) clearance over time at TRHs and CHs. The secondary outcome was intraoperative and postoperative complications, stone clearance and mortality with postoperative rendezvous ERCP compared to intraoperative rendezvous ERCP. The intraoperative complications analyzed were overall complications and bleeding and the postoperative complications included overall complications, bleeding, pancreatitis, cholangitis, bile leakage, infection with abscess, percutaneous drainage, antibiotic treatment, ICU stay, readmission and reoperation within 30 days (as a proxy for stone clearance rate/retained stones) and 30-day mortality. We have also analyzed length of hospital stay.

The Regional Ethics Review Board in Uppsala approved the study the 18th of September 2018 (Reference Number: 2016/281/1) after a complementary application to the original ethics approval from 2nd of November 2016 (Reference Number: 2016/281/1).

### Statistics

Univariate and multivariate regression analyzes were used as well as Pearson Chi Square Test and Student’s *T* Test.

The analyzes were based on patients undergoing cholecystectomy with intraoperative ERCP and patients undergoing cholecystectomy as well as postoperative ERCP in two separate procedures. The complication rate was determined by extracting intraoperative and postoperative complications within 30 days after the cholecystectomy as well as the postoperative ERCP. In univariate and multivariate logistic regression analysis, the odds ratio for intra- and postoperative complications was determined, adjusted for gender, age and ASA score.

Statistical significance was defined as p < 0.05. Statistical analysis was carried out using JMP^®^ Pro version 14.0.0 (SAS Institute Inc., USA).

## Results

**Fig. 3:**
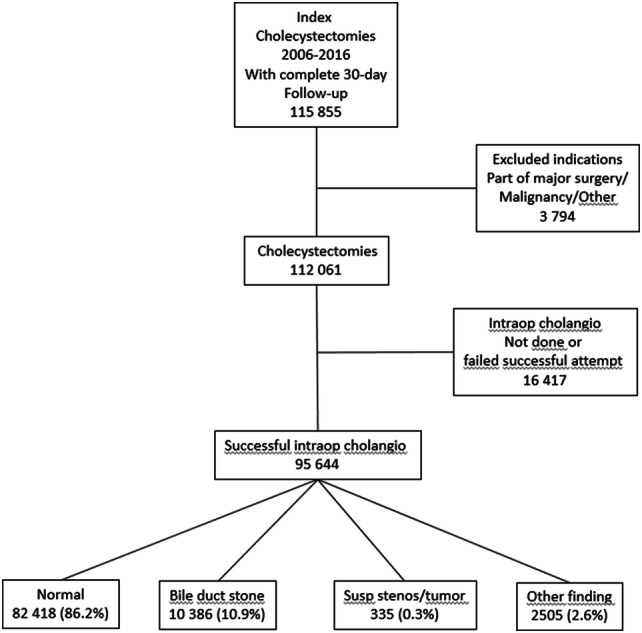
Flowchart cholecystectomies in Sweden 2006–2016

**Fig. 4:**
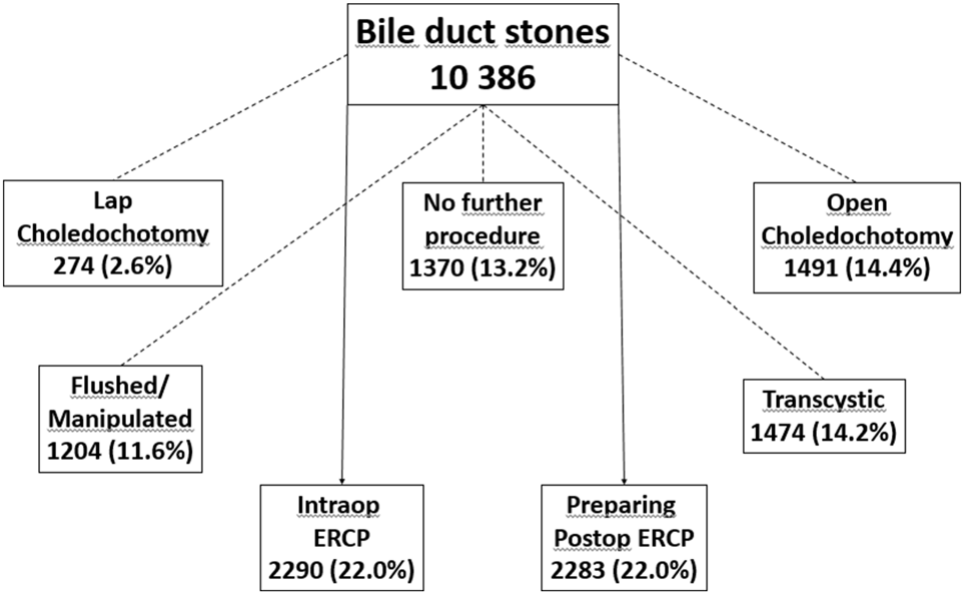
Flowchart common bile duct stones in Sweden 2006–2016

In this study all cholecystectomies performed 2006–2016, where CBDS were found at intraoperative cholangiography and 30-day follow-up was complete were included. In total 10,386 procedures fulfilled the criteria (Fig. 3). Data for CBDS clearance and complications were retrieved for intraoperative rendezvous ERCP (n = 2290) as well as for procedures where preparation for postoperative rendezvous ERCP was undertaken (n = 2283, Fig. 4).

Patients in the group with preparation for postoperative rendezvous ERCP were slightly older. More patients in the intraoperative ERCP group had an ASA score > 2. There were no gender-specific differences between the two groups (Table 1).

**Table 1:** Demographics of the two groups: intraoperative and preparing postoperative ERCP, ERCP within 30 days

	ERCP		*P*
	Intraop n = 2290	Preparing postop n = 2283	
Females^a^			
n	**1493**	**1536**	
%	65.2	67.3	
Males			
n	**797**	**747**	
%	34.8	32.7	
Total			
n	**2290**	**2283**	0.1363
ASA 1-2			
n	**2071**	**2104**	
%	90.4	92.2	
ASA > 2			
n	**219**	**179**	
%	9.6	7.8	**0.0388**
Age^b^ (years)			
Mean	**51.3**	**52.9**	
SEM	0.4	0.4	**0.0023**

Statistically significant values are given in bold

^a^Pearson ChiSquare

^b^Student’s *t* test

The percentage intraoperative ERCP procedures increased from 7.5 to 43.1% during the study period, and since 2013 it has been the predominant method for management of CBDS found at cholecystectomy. Preparation for postoperative rendezvous ERCP, on the other hand, gradually decreased during the final years of the study period, 21.2% in 2006 and 17.2% in 2016 (Fig. 1).

Management of CBDS differ between TRHs and CHs. The most commonly used method was intraoperative ERCP, though this option was more commonly used at TRHs; 47.8% (2016) compared to 41.8% at CHs. On the other hand, preparation for postoperative rendezvous ERCP was more frequent in CHs; 19.7% (2016) compared to 8.3% at TRHs (Fig. 2).

The intraoperative complication rate was lower in the intraoperative rendezvous ERCP group compared to the postoperative rendezvous ERCP group (2.0% vs. 3.4%; p = 0.0031). The same pattern was noted regarding postoperative complication rates (15.6% vs. 21.8%; p < 0.0001).

Intraoperative bleeding rate was lower in the intraoperative rendezvous ERCP group compared to the postoperative ERCP rendezvous group (0.4% vs. 1.1%; p = 0.0106).

There were no significant differences between the two groups regarding postoperative bleeding, pancreatitis, cholangitis, percutaneous drainage, antibiotic treatment, ICU stay or reoperation within 30 days.

Postoperative bile leakage and infection with abscess rates were lower in the intraoperative ERCP group compared to the postoperative rendezvous ERCP group (1.4% vs. 2.7%; p = 0.0025 and 1.9% vs. 2.9%; p = 0.0197 respectively).

Readmission rate within 30 days and 30-day mortality were higher in the intraoperative ERCP group (0.7% vs. 0.3%; p = 0.0498 and 0.31% vs. 0.04%; p = 0.0341 respectively) (Table 2).

**Table 2:** Intra- and postoperative complications n (%), ERCP within 30 days

	Intraop ERCP n (%)	Preparing postop n (%)	*P*^a^
Intraoperative complications			
Overall	45 (2.0)	77 (3.4)	**0.0031**
Bleeding	10 (0.4)	25 (1.1)	**0.0106**
Postoperative complications			
Overall	357 (15.6)	497 (21.8)	**<0.0001**
Bleeding	28 (1.2)	20 (0.9)	0.2501
Pancreatitis	108 (4.7)	101 (4.4)	0.6362
Cholangitis	14 (0.6)	21 (0.9)	0.2314
Bile leakage	33 (1.4)	62 (2.7)	**0.0025**
Infection with abscess	43 (1.9)	67 (2.9)	**0.0197**
Percutaneous drainage	51 (2.2)	69 (3.0)	0.0925
Antibiotic treatment	223 (9.7)	237 (10.4)	0.4697
ICU stay	6 (0.3)	3 (0.1)	0.3191
Readmission within 30 days	15 (0.7)	6 (0.3)	**0.0498**
Reop within 30 days	46 (2.0)	48 (2.1)	0.8232
Mortality 30 days	7 (0.31)	1 (0.04)	**0.0341**

Statistically significant values are given in bold

^a^Pearson ChiSquare

In the multivariate analzses overall intraoperative and overall postoperative complications, intraoperative bleeding, postoperative bile leakage and postoperative infection with abscess were all significantly higher in the postoperative rendezvous ERCP group. The Odds Ratio for overall complications in the postoperative rendezvous ERCP group with the intraoperative ERCP group as a reference was 1.69 (CI 1.16–2.45) intraoperatively, and 1.50 (CI 1.29–1.75) postoperatively. The odds ratio for intraoperative bleeding was 2.46 (CI 1.17–5.16), for postoperative bile leakage 1.89 (CI 1.23–2.90) and for postoperative infection with abscess 1.55 (CI 1.05–2.29) (Table 3).

**Table 3:** Intra- and postoperative complications of preparing for postoperative versus intraoperative ERCP (reference)

	Intraop ERCP ref		
	OR	95% CI	*P*
Intraoperative complications			
Overall	1.69	(1.16–2.45)	**0.0061**
Bleeding	2.46	(1.17–5.16)	**0.0170**
Postoperative complications			
Overall	1.50	(1.29–1.75)	**< 0.0001**
Bleeding	0.72	(0.40–1.28)	0.2581
Pancreatitis	0.95	(0.72–1.25)	0.7053
Cholangitis	1.53	(0.77–3.02)	0.2229
Bile leakage	1.89	(1.23–2.90)	**0.0034**
Infection with abscess	1.55	(1.05–2.29)	**0.0270**
Percutaneous drainage	1.34	(0.93–1.94)	0.1191
Antibiotic treatment	1.06	(0.88–1.29)	0.5336
ICU stay	0.51	(0.13–2.04)	0.3394
Readmission within 30 days	0.41	(0.16–1.07)	0.0681
Reop within 30 days	1.05	(0.70–1.58)	0.8146
Mortality 30 days	0.16	(0.02–1.35)	0.0927

ERCP within 30 days. Multivariate analysis

Adjusted for gender, age and ASA

Statistically significant values are given in bold

The total length of hospital stay was somewhat shorter for patients who underwent intraoperative ERCP compared to patients who were prepared for postoperative ERCP (Table 4).

**Table 4:** Length of stay (days)

Intraop ERCP		Preparing postop ERCP		
Mean	SEM	Mean	SEM	*p*
4.7	0.1	5.1	0.1	0.0454

## Discussion

In this study, based on prospectively assembled population-based data from GallRiks, we compared methods of managing CBDS found at intraoperative cholangiography in a large number of cholecystectomies over a long period of time. We surveyed the management of CBDS over time as well as differences between tertiary referral hospitals compared to community/county hospitals. The decision on treatment regimens is mainly based on local traditions at each respective hospital. There are units where cholecystectomies are performed on regular basis but where there is a lack of ERCP resources. At such units, two-stage procedures are the only choice besides transcystic stone extraction or extraction by choledochotomy.

We focused on the two most common treatment options regarding choledocholithiasis; intraoperative and postoperative rendezvous ERCP and compared these methods regarding intraoperative and postoperative complication rates as well as readmission, reoperation and mortality. In recent years intraoperative rendezvous ERCP has been established as the method of choice in many units where ERCP resources at cholecystectomy are available. The result of this is that it has not been possible to conduct a prospective randomized-controlled trial comparing the two methods.

CBDS are commonly found during cholecystectomy when intraoperative cholangiography (IOC) is routinely performed [15]. Open choledochotomy, traditionally considered the first-hand technique for managing CBDS, has decreased in recent years. On the other hand, minimally invasive laparoscopic and laparo-endoscopic methods have become more frequently used. There are several strategies to manage CBDS but the optimal method as well as timing is still under debate [5, 16, 23–30].

ERCP is a well-established method for treatment of diseases of the common bile ducts, including bile duct calculi [1, 31]. ERCP has traditionally been performed as a two-stage procedure, either as preoperative ERCP followed by laparoscopic cholecystectomy or laparoscopic cholecystectomy followed by postoperative ERCP. However, 4–18% of attempted ERCPs fail due to inability to cannulate the bile duct [1, 32]. ERCP may also lead to serious complications, of which post-ERCP pancreatitis (PEP) is the most frequent with an incidence of 3.5–5% [1, 33]. The risk of developing PEP depends on patient-related factors such as female gender, younger age and Sphincter of Oddi dysfunction [34]. Technical factors such as manipulation of and injection of contrast into the pancreatic duct, biliary balloon sphincter dilation and cannulation attempts lasting > 5 min, also increase the risk [33, 35, 36].

Intraoperative rendezvous ERCP is an effective and safe method to treat CBDS found at cholecystectomy and concomitant cholangiography [7–14]. The operative technique of laparo-endoscopic rendezvous is straight-forward and suitable for almost all patients with CBDS. In this way cholecystectomy and management of CBDS are performed in a single procedure, thereby limiting anesthesia to one occasion and minimal hospital stay, health care resources and costs. In Sweden, therefore, at hospitals where ERCP is available, intraoperative rendezvous ERCP has been the method of choice in the management of CBDS during cholecystectomy.

In this study we have shown that during the period 2006–2016, intraoperative ERCP gradually became the predominating method to manage CBDS at all hospital levels in Sweden and by 2016 60% of patients were managed this way. Intraoperative rendezvous ERCP was the method of choice at all hospital levels, but most commonly used in TRHs. Preparing for postoperative rendezvous ERCP, on the other hand, was performed twice as often in CHs compared to TRHs. In 2016 postoperative rendezvous ERCP was the second most common method of managing CBDS in these hospitals compared to only the fourth most common method at TRHs, probably due to a lack of resources for performing intraoperative ERCP in non-specialized centers.

The complication rate regarding intraoperative ERCP and preparing postoperative ERCP is assessed intraoperatively (overall complications, bleeding) as well as 30 days after the procedure (overall complications, bleeding, pancreatitis, cholangitis, bile leakage, infection with abscess, percutaneous drainage, antibiotic treatment, ICU stay, readmission, reoperation, mortality). Since intraoperative ERCP is conducted simultaneously with the cholecystectomy and postoperative ERCP in most cases is performed within 1 or 2 days after cholecystectomy we cannot exclude that some of the observed complications could have been caused by the cholecystectomy rather than the ERCP.

The overall incidence of intra- and postoperative complications as well as intraoperative bleeding, postoperative bile leakage and postoperative infection with abscess was higher in postoperative rendezvous ERCP compared to intraoperative rendezvous ERCP. Manipulation of the guidewire preparing for postoperative ERCP could be one possible explanation for a higher rate of postoperative bile leakage and infection in this group. If the clips around the cystic duct, anchoring the guide wire, are applied too loose there probably is a risk for subsequent bile leakage.

The rate of the most common surgical complication, post-ERCP pancreatitis, was not significantly higher in patients treated with postoperative rendezvous ERCP, neither were postoperative bleeding, cholangitis, need for percutaneous drainage, antibiotic treatment, ICU stay or 30-day mortality.

Readmission and reoperation within 30 days rates, a proxy for stone clearance and effectiveness of the ERCP procedure, were also similar between the groups.

Since many cholecystectomies are performed in hospitals where ERCP is not performed at all, or performed during off-hours when access to ERCP is limited, there is a need for an alternative management solution. Preparing for postoperative rendezvous ERCP by leaving a guidewire for later definitive treatment of CBDS the days following cholecystectomy is a feasible alternative. The routine of leaving a guidewire through the abdominal wall bandaged to the skin cause some discomfort to the patient, even if most patients seem to tolerate the guidewire quite well.

Based on the results of this study we believe that laparo-endoscopic biliary duct stone clearance techniques are safe and effective. Intraoperative rendezvous ERCP is the method of choice due to a lower complication rate and optimal utilization of hospital resources. Postoperative rendezvous ERCP constitutes an acceptable alternative in situations where ERCP resources are lacking or limited. It is technically easier to perform compared to non-rendezvous postoperative ERCP since the cannulation of the common bile duct is facilitated by a guide wire to the duodenum. This often renders the ERCP procedure faster, with less risk of traumatizing the papilla with subsequent oedema and in some cases PEP.
